# Helminths of three species of opossums (Mammalia, Didelphidae) from Mexico

**DOI:** 10.3897/zookeys.511.9571

**Published:** 2015-07-02

**Authors:** Karla Acosta-Virgen, Jorge López-Caballero, Luis García-Prieto, Rosario Mata-López

**Affiliations:** 1 Departamento de Biología Evolutiva, Facultad de Ciencias, Universidad Nacional Autónoma de México, C.P.04510, Mexico City, Mexico; 2 Colección Nacional de Helmintos, Instituto de Biología, Universidad Nacional Autónoma de México, CP 04510, Mexico City, Mexico; 3 Posgrado en Ciencias Biológicas, Universidad Nacional Autónoma de México, Apartado 70-153, C.P. 04510, Mexico City, Mexico

**Keywords:** Didelphidae, *Didelphis virginiana*, *Didelphis marsupialis*, *Philander opossum*, parasites

## Abstract

From August 2011 to November 2013, 68 opossums (8 *Didelphis* sp., 40 *Didelphis virginiana*, 15 *Didelphis marsupialis*, and 5 *Philander opossum*) were collected in 18 localities from 12 Mexican states. A total of 12,188 helminths representing 21 taxa were identified (6 trematodes, 2 cestodes, 3 acanthocephalans and 10 nematodes). Sixty-six new locality records, 9 new host records, and one species, the trematode *Brachylaima didelphus*, is added to the composition of the helminth fauna of the opossums in Mexico. These data, in conjunction with previous records, bring the number of taxa parasitizing the Mexican terrestrial marsupials to 41. Among these species, we recognized a group of helminths typical of didelphids in other parts of the Americas. This group is constituted by the trematode *Rhopalias coronatus*, the acanthocephalan *Oligacanthorhynchus microcephalus* and the nematodes *Cruzia tentaculata*, *Gnathostoma turgidum*, and *Turgida turgida*. In general, the helminth fauna of each didelphid species showed a stable taxonomic composition with respect to previously sampled sites. This situation suggests that the rate of accumulation of helminth species in the inventory of these 3 species of terrestrial marsupials in the Neotropical portion of Mexico is decreasing; however, new samplings in the Nearctic portion of this country will probably increase the richness of the helminthological inventory of this group of mammals.

## Introduction

Less than 25% of the 525 species of mammals distributed in Mexico have been examined for helminth parasites (García-Prieto et al. 2012). To date, 336 nominal taxa of helminths have been recorded in mammals, 26 associated with 3 species of terrestrial opossums (Virginia opossum, *Didelphis virginiana* Kerr, the common opossum *Didelphis marsupialis* Linnaeus, and the Gray four-eyed opossum *Philander opossum* Linnaeus) from this country. However, the knowledge of the helminth richness associated with this host group is incomplete due to the wide distribution of these mammals in Mexico. *Didelphis marsupialis* occurs from Tamaulipas State and west San Luis Potosí until the Yucatán peninsula. *Didelphis virginiana* inhabits almost all of Mexico, except for the central Plateau and Baja California peninsula. *Philander opossum* occurs from south Tamaulipas State along the Gulf of Mexico coast and Chiapas State (Arcangeli-Álvarez 2010, Cervantes et al. 2010). The main objective of this work is to present new records of helminth species parasitizing these 3 species of opossums in Mexico and to compare the finding to previous records.

## Materials and methods

From August 2011 to November 2013, 68 opossums (8 *Didelphis* sp., 40 *Didelphis virginiana*, 15 *Didelphis marsupialis*, and 5 *Philander opossum*) were collected in 18 localities from 12 Mexican states (Table 1), under the collecting permit FAUT 0057 issued by the Secretaría del Medio Ambiente y Recursos Naturales (SEMARNAT), Mexico. Mammals were shot by local hunters or caught with Tomahawk traps and then killed with intraperitoneal sodium pentobarbital overdose. Opossums were dissected within the following 4 h. and all organs were examined under a stereomicroscope. Helminths were placed in Petri dishes with 0.85% saline solution. Platyhelminths and nematodes were fixed with hot 4% formalin and preserved in 70% ethanol; acanthocephalans were chilled in distilled water for 10–12 h. Once the proboscis was everted, they were preserved in 70% ethanol. Platyhelminths and acanthocephalans were stained with Mayer’s paracarmin, cleared with methyl salicilate, and mounted in Canada balsam. Nematodes were cleared using Amman’s lactophenol and temporarily mounted for morphological study (Lamothe-Argumedo 1997). Voucher specimens of all helminth species were deposited at Colección Nacional de Helmintos (CNHE), Instituto de Biología, Universidad Nacional Autónoma de México, Mexico City.

**Table 1.** Sampling sites for opossum species analyzed in this study.

State	Locality^†^/collection date (month/year)	Geographic coordinates	Sample size/host species	Altitude (easl)
Campeche	Escárcega^1^ 07/2012	18°37'00"N; 90°43'13”W	3/*Didelphis virginiana*; 1/*Didelphis marsupialis*	82
Chiapas	Agua Fría^2^ 06/2012; 03/2013	16°15'26"N; 93°53'55"W	5/*Didelphis* sp.; 1/*Didelphis virginiana*	60
	Finca Brasil^3^ 06/2012	15°05'41"N; 92°13'45"W	2/*Didelphis* sp.; 3/*Didelphis virginiana*; 2/*Didelphis marsupialis*	463
Colima	Coquimatlán^4^ 09/2012	19°10'28"N; 103°50'39”W	6/*Didelphis virginiana*	550
Distrito Federal	Pedregal de San Ángel^5^ 02/2014	19°19'14"N; 99°12'33"W	2/*Didelphis virginiana*	2268
Guanajuato	Rincón de Martínez^6^ 02/2013	20°19'44"N; 101°34'42”W	2/*Didelphis virginiana*	1730
Hidalgo	Tianguistengo^7^ 03/2014	19°10'50"N; 99°28'06"W	2/*Didelphis virginiana*	2620
Morelos	Tepoztlán^8^ 08/2014	19°00'07"N; 99°06'00"W	1/*Didelphis virginiana*	1700
Oaxaca	Cerro del Tepezcuintle^9^ 08/2013	18°15'28”N; 96°24'00"W	2/*Didelphis virginiana*	87
Puebla	Coapan^10^ 08/2014	18°25'42"N; 97°24'30"W	1/*Didelphis* sp.; 1/*Didelphis virginiana*	1648
	Zapotitlán Salinas^11^ 08/2014	18°19'45"N; 97°28'30"W	1/*Didelphis virginiana*	2240
Tabasco	Teapa^12^ 06-07/2013	17°33'59"N; 92°57'00”W	2/*Didelphis virginiana* 1/*Didelphis marsupialis*	72
	Villahermosa^13^ 01/2012	17°34'17”N; 92°57'09”W	3/*Didelphis virginiana*	10
Veracruz	Tlacotalpan^14^ 02/2012	18°37'40”N; 95°40'40”W	2/*Didelphis virginiana*; 8/*Didelphis marsupialis*; 3/*Philander opossum*	10
	Los Tuxtlas^14^ 08/2011; 03/2012	18°34'21”N; 95°04'30”W	3/*Didelphis virginiana*; 3/*Didelphis marsupialis* 2/*Philander opossum*	300
Yucatán	Mérida^15^ 11/2013	20°58'04"N; 89°37'18"W	5/*Didelphis virginiana*	16
	Tzucacab^16^ 11/2013	20°00'58"N; 89°01'12"W	1/*Didelphis marsupialis*	36

† The superscript numbers indicate the position of the localities in the Figure 1.

## Results

**Figure 1.**
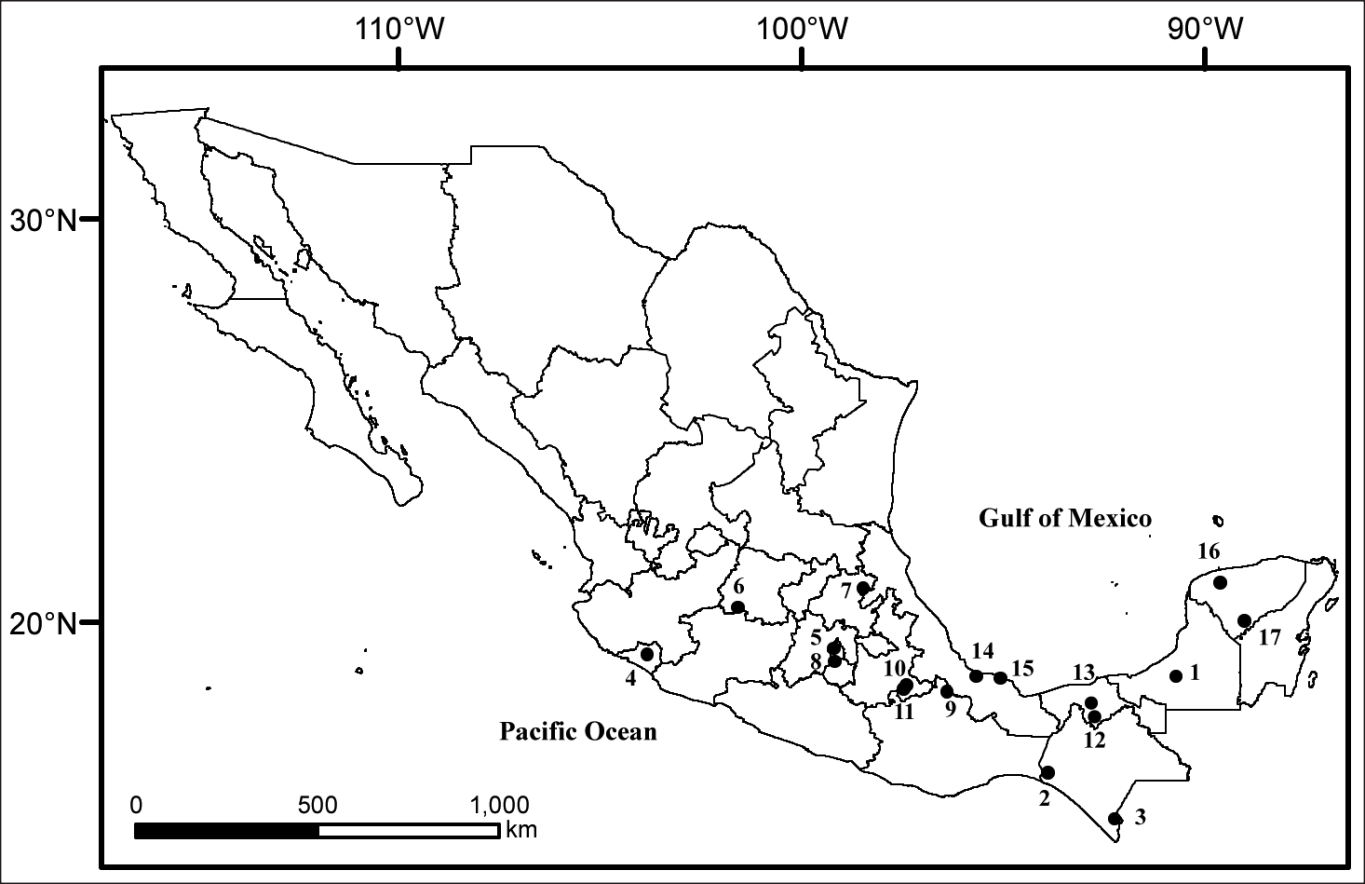
Map of Mexico showing the sampled localities in the present study.

A total of 12,188 helminths representing 21 taxa were identified in the 68 opossums collected from 18 localities within 12 states of Mexico (Figure 1). Six trematode, 2 cestode, 3 acanthocephalan, and 10 nematode species were collected. Below, we present a checklist of the helminth species recorded, indicating the site of infection, current records with State and locality where the hosts were collected, host species, CNHE accession numbers, and previous records from Mexico.

### Parasite-Host list

† New locality record; ‡ New record for Mexico; * New host in Mexico.

**Phylum Platyhelminthes Gegenbaur, 1859**

**Class Trematoda Rudolphi, 1808**

**Family Opisthorchiidae Braun, 1901**

***Amphimerus caudalitestis* Caballero, Grocott & Zerecero, 1952**

**Site of infection.** Gall-bladder.

**Present records.** VERACRUZ: Los Tuxtlas: Didelphis marsupialis*, Didelphis virginiana*.

**Specimens deposited.** CNHE 9481–2.

**Previous records in Mexico.** VERACRUZ: Los Tuxtlas: *Philander opossum* (Cañeda-Guzmán 1997).

**Remarks.** These specimens belong to *Amphimerus caudalitestis* due to the position of the reproductive organs and the separation of the vitelline glands in two fields lying anterior and posterior to the ovary. Furthermore, the uterus has a zig-zag shape, occupying intercecal extension and the S-shape of the excretory vesicle, sinuous between both testes (Caballero et al. 1952).

***Brachylaima didelphus* Premvati & Bair, 1979^‡^**

**Site of infection.** Intestine.

**Present records.** CAMPECHE: Escárcega^†^: Didelphis virginiana*.

**Specimens deposited.** CNHE 9483–4.

**Remarks.** The specific identification of this material follows Premvati and Blair (1979) and is based on the disposition of the vitellaria which extending from pharynx to posterior end.

**Family Phaneropsolidae Mehra, 1935**

***Philandrophilus magnacirrus* Thatcher, 1970**

**Site of infection.** Gall-bladder.

**Present records.** Los Tuxtlas: Didelphis marsupialis*, *Philander opossum*.

**Specimens deposited.** CNHE 9485–6.

**Previous records in Mexico.** VERACRUZ: Los Tuxtlas: *Philander opossum* (Cañeda-Guzmán 1997).

**Remarks.** In accordance with Thatcher (1970) this species is characterised by having body flattened and pyriform, covered with small spines. Cirrus and cirrus sac large. Parasites in gall-bladder of marsupials.

**Family Rhopaliidae Looss, 1899**

***Rhopalias caballeroi* Kifune & Uyema, 1982**

**Site of infection.** Intestine.

**Present records.** VERACRUZ: Tlacotalpan^†^: Philander opossum*.

**Specimens deposited.** CNHE 9487.

**Previous records in Mexico.** VERACRUZ: Los Tuxtlas: *Didelphis* sp. (Haverkost and Gardner 2008).

**Remarks.** *Rhopalias caballeroi* is distinguished by the absence of oral and flanking spines, and because it has between 4 and 11 spines visible within tentacle sacs (Haverkost and Gardner 2008).

***Rhopalias coronatus* (Rudolphi, 1819)**

**Site of infection.** Intestine.

**Present records.** CHIAPAS: Agua Fría^†^: *Didelphis marsupialis*, *Didelphis virginiana*, *Didelphis* sp., *Philander opossum*; Finca Brasil^†^: *Didelphis marsupialis*, *Didelphis virginiana*, *Philander opossum*. OAXACA: Cerro del Tepezcuintle^†^, San Miguel Soyaltepec^†^: *Didelphis virginiana*. TABASCO: Cunduacán^†^: *Didelphis virginiana*; Grutas de Coconá^†^, Teapa^†^: *Didelphis marsupialis*. VERACRUZ: Los Tuxtlas: *Didelphis marsupialis*, *Didelphis virginiana*, *Philander opossum*; Tlacotalpan^†^: *Didelphis marsupialis*, *Didelphis* sp. YUCATÁN: Mérida^†^: *Didelphis marsupialis*.

**Specimens deposited.** CNHE 9488–9504.

**Previous records in Mexico.** CHIAPAS: Motozintla: *Didelphis* sp. (Caballero et al. 1944). NUEVO LEÓN: Colonia Country La Silla, Huinala, Los Lirios: *Didelphis marsupialis* (Romero 1981). OAXACA: Cuicatlán: *Didelphis* sp. (Pérez-Ponce de León et al. 2007). QUINTANA ROO: Rancho La Ceiba: *Didelphis marsupialis* (Kingston and Tai 1968). VERACRUZ: Los Tuxtlas: *Didelphis marsupialis*, *Didelphis virginiana*, *Philander opossum* (Cañeda-Guzmán 1997), *Didelphis* sp. (Haverkost and Gardner 2008); Alvarado: *Didelphis virginiana* (Monet-Mendoza et al. 2005).

**Remarks.** The diagnostic traits of this species are: flanking and oral spines present. Between 3 and 11 spines visible within tentacle sacs, which extend far beyond the posterior margin of the pharynx (Haverkost and Gardner 2008).

***Rhopalias macracanthus* Chandler, 1932**

**Site of infection.** Intestine.

**Present records.** VERACRUZ: Los Tuxtlas: *Didelphis marsupialis*, *Didelphis* sp., *Didelphis virginiana*; Tlacotalpan^†^: *Didelphis marsupialis*, *Philander opossum*.

**Specimens deposited.** CNHE 9505–9.

**Previous records in Mexico.** COLIMA: Comala: *Didelphis marsupialis* (Lamothe-Argumedo 1978); La Esperanza: *Didelphis marsupialis* (Miyazaki et al. 1980). CHIAPAS: Jaltenango: *Didelphis* sp. (Caballero 1946); Motozintla: *Didelphis* sp. (Caballero et al. 1944); Pueblo Nuevo (Pérez-Ponce de León et al. 2007). OAXACA: Carretera Temascal-Tuxtepec: *Didelphis virginiana* (Monet-Mendoza et al. 2005). QUINTANA ROO: Rancho La Ceiba: *Didelphis marsupialis* (Kingston and Tai 1968). VERACRUZ: Alvarado: *Didelphis virginiana* (Monet-Mendoza et al. 2005); Los Tuxtlas: *Didelphis marsupialis*, *Didelphis virginiana*, *Philander opossum* (Cañeda-Guzmán 1997), *Didelphis* sp. (Haverkost and Gardner 2008).

**Remarks.** This species was identified by having tentacle sacs that do not extend beyond the posterior margin of the pharynx and by having only flanking spines (Haverkost and Gardner 2008).

**Class Eucestoda Southwell, 1930**

**Family Anoplocephalidae Cholodkovsky, 1902**

***Mathevotaenia* sp.**

**Site of infection.** Intestine.

**Present records.** COLIMA: Colima: *Didelphis virginiana*.

**Specimens deposited.** CNHE 9514.

**Previous records in Mexico.** CHIAPAS: Lagos de Colón: *Didelphis virginiana* (Monet-Mendoza et al. 2005). COLIMA: Colima: *Didelphis virginiana* (García-Prieto et al. 2012).

**Remarks.** This material represents a new species which will be described separately.

**Family Proteocephalidae La Rue, 1911**

***Thaumasioscolex didelphidis* Cañeda-Guzmán, de Chambrier & Scholz, 2001**

**Site of infection.** Intestine.

**Present records.** CHIAPAS: Finca Brasil^†^: Didelphis virginiana*, *Didelphis marsupialis*.

**Specimens deposited.** CNHE 9528.

**Previous records in Mexico.** VERACRUZ: Los Tuxtlas: *Didelphis marsupialis* (Cañeda-Guzmán et al. 2001).

**Remarks.** In accordance with Cañeda-Guzmán et al. (2001), *Thaumasioscolex didelphidis* is distinguished by the morphology of the scolex that is formed by 4 well separated lobes each containing 1 noncircular sucker opening laterally inside the exterolateral cavity, a large-sized body and by the shape of gravid proglottids that are inversely craspedote, among others.

**Phylum Acanthocephala (Rudolphi, 1808)**

**Family Oligacanthorhynchidae Southwell & Macfie, 1925**

***Oligacanthorhynchus microcephalus* (Rudolphi, 1819) Schmidt, 1972**

**Site of infection.** Intestine.

**Present records.** HIDALGO: Tianguistengo^†^: *Didelphis virginiana*.

**Specimens deposited.** CNHE 9510.

**Previous records in Mexico.** CAMPECHE: Escárcega: *Didelphis marsupialis*, *Didelphis virginiana* (López-Caballero et al. 2015). COLIMA: Tecomán: *Didelphis virginiana* (García-Prieto et al. 2010). CHIAPAS: Agua Fría: *Didelphis marsupialis*, *Didelphis virginiana*, *Philander opossum* (López-Caballero et al. 2015); Cascadas de Agua Azul: *Didelphis virginiana* (Prado-Ancona 1993); Finca Brasil: *Didelphis marsupialis*, *Didelphis virginiana*, *Philander opossum* (López-Caballero et al. 2015). MICHOACÁN: Agua Blanca: *Didelphis virginiana* (Prado-Ancona 1993). GUANAJUATO: Rincón de Martínez: *Didelphis virginiana* (López-Caballero et al. 2015). MORELOS: Progreso: *Didelphis virginiana* (García-Prieto et al. 2010). OAXACA: Soyaltepec: *Didelphis virginiana* (López-Caballero et al. 2015); Temascal: *Didelphis virginiana* (García-Varela et al. 2000). TABASCO: Cunduacán: *Didelphis virginiana* (López-Caballero et al. 2015); Ranchería el Boquerón: *Didelphis marsupialis* (García-Prieto et al. 2010); Río Oxolotán: *Philander opossum* (Prado-Ancona 1993). VERACRUZ: Los Tuxtlas: *Didelphis marsupialis*, *Didelphis virginiana*, *Philander opossum* (Prado-Ancona 1993; Cañeda-Guzmán 1997); Tlacotalpan: *Didelphis virginiana* (López-Caballero et al. 2015). YUCATÁN: Mérida: *Didelphis marsupialis*, *Didelphis virginiana* (López-Caballero et al. 2015).

**Remarks.** With the exception of records made by López-Caballero et al. (2015) all other previous records were listed as *Oligacanthorhynchus tortuosa*, but this species is a junior synonym of *Oligacanthorhynchus microcephalus* (Richardson et al. 2014). The hook and cement gland number (36 and 8, respectively), as well as the eggs size (0.83-0.110 X 0.38-0.50) are considered as diagnostic traits of this species by López-Caballero et al. (2015).

***Oncicola luehei* (Travassos, 1917) Schmidt, 1972**

**Site of infection.** Intestine.

**Present records.** VERACRUZ: Los Tuxtlas: Didelphis marsupialis*.

**Specimens deposited.** CNHE 9511–12.

**Previous records in Mexico.** VERACRUZ: Los Tuxtlas: *Didelphis virginiana* (Prado-Ancona 1993; Cañeda-Guzmán 1997).

**Remarks**. These specimens belong to *Oncicola luehei* because the dimensions of the proboscis, the number of hooks (36), as well as its size and arrangement fits to the morphology mentioned by Machado (1950).

**Family Plagiorhynchidae Golvan, 1960**

***Porrorchis nickoli* Salgado-Maldonado & Cruz-Reyes, 2002**

**Site of infection.** Intestine.

**Present records.** VERACRUZ: Los Tuxtlas: *Didelphis virginiana*.

**Specimens deposited.** CNHE 9513.

**Previous records in Mexico.** CHIAPAS: Cascadas de Agua Azul: *Didelphis virginiana* (Salgado-Maldonado and Cruz-Reyes 2002). TABASCO: Río Oxolotán: *Philander opossum* (Salgado-Maldonado and Cruz-Reyes 2002). VERACRUZ: Lago de Catemaco, Sontecomapan: *Didelphis virginiana* (Salgado-Maldonado and Cruz-Reyes 2002); Martínez de la Torre: *Didelphis marsupialis* (Salgado-Maldonado and Cruz-Reyes 2002); Los Tuxtlas: *Didelphis marsupialis*, *Didelphis virginiana*, *Philander opossum* (Salgado-Maldonado and Cruz-Reyes 2002).

**Remarks**. According to Salgado-Maldonado and Cruz-Reyes (2002), three characteristics diagnosed this acanthocephalan species: (1) a smaller proboscis, (2) the armature of proboscis bearing few rows and few hooks per row compared with other species, and (3) the male reproductive system occupying only the posterior half of trunk.

**Phylum Nematoda Rudolphi, 1808**

**Family Metastrongylidae Leiper, 1912**

***Didelphostrongylus* hayesi Prestwood, 1976**

**Site of infection.** Lungs.

**Present records.** DISTRITO FEDERAL: Pedregal de San Ángel^†^: *Didelphis virginiana*; GUANAJUATO: Irapuato^†^: *Didelphis virginiana*^†^. HIDALGO: Tianguistengo^†^: *Didelphis virginiana*. MORELOS: Tepoztlán^†^: *Didelphis virginiana*.

**Specimens deposited.** CNHE 8969, 9024, 9554–9556, 9562.

**Previous records in Mexico.** COLIMA: ND: *Didelphis virginiana* (García-Márquez et al. 2012). GUERRERO: Laguna de Tres Palos, Taxco: *Didelphis virginiana* (Monet-Mendoza et al. 2005). OAXACA: Temascal: *Didelphis virginiana* (Monet-Mendoza et al. 2005).

**Remarks**. Our material was identified following Prestwood (1976); this species is characterised because the oral opening is surrounded by lips, the morphology and size of the spicules and the number and arrangement of bursal rays.

**Family Aspidoderidae Skrjabin & Schikhobalova, 1947**

***Aspidodera raillieti* Travassos, 1913**

**Site of infection.** Intestine.

**Present record.** TABASCO: Villahermosa^†^: Didelphis virginiana*. VERACRUZ: Los Tuxtlas^†^: *Didelphis virginiana*, Philander opossum*.

**Specimens deposited.** CNHE 8971–3.

**Previous records in Mexico.** CHIAPAS: Motozintla: *Didelphis* sp. (Caballero and Zerecero 1944).

**Remarks.** These specimens were identified based on Jiménez-Ruiz et al. (2006) and compared with further description of the species made by Chagas-Moutinho et al. (2014). *Aspidodera raillieti* can be distinguished because the cephalic cordons exceed the level of the oral vestibule and touch the base of cephalic cap, as well as by having a digitiform projection on the left ventrolateral oral lip.

**Family Kathlanidae Lane, 1914**

***Cruzia tentaculata* (Rudolphi, 1819) Travassos, 1917**

**Site of infection.** Caecum.

**Present records.** CAMPECHE: Escárcega^†^: *Didelphis marsupialis*, *Didelphis virginiana*. CHIAPAS: Arriaga^†^: *Didelphis* sp., *Didelphis virginiana*; Tapachula^†^: *Didelphis* sp., *Didelphis marsupialis*, *Didelphis virginiana*. COLIMA: Colima^†^: *Didelphis virginiana*. DISTRITO FEDERAL: Pedregal de San Ángel^†^: *Didelphis virginiana*. GUANAJUATO: Irapuato^†^: *Didelphis virginiana*. HIDALGO: Tianguistengo^†^: *Didelphis virginiana*. MORELOS: Tepoztlán^†^: *Didelphis virginiana*. OAXACA: Soyaltepec^†^: *Didelphis virginiana*. PUEBLA: Carretera Coapan-Huajuapan de León^†^: *Didelphis* sp.; Coapan^†^: *Didelphis virginiana*; Zapotitlán Salinas^†^: *Didelphis virginiana*. TABASCO: Teapa^†^: *Didelphis marsupialis*; Villahermosa^†^: *Didelphis virginiana*. VERACRUZ: Los Tuxtlas: *Didelphis marsupialis*, *Didelphis virginiana*, *Philander opossum*; Tlacotalpan^†^: *Didelphis marsupialis*, *Didelphis virginiana*, *Philander opossum*. YUCATÁN: Mérida^†^: *Didelphis virginiana*; Tzucacab^†^: *Didelphis marsupialis*.

**Specimens deposited.** CNHE 8999, 9000–17, 9533–9540, 9557, 9563.

**Previous records in Mexico.** CHIAPAS: Motozintla: *Didelphis* sp. (Caballero and Zerecero 1944); Jaltenango: *Didelphis marsupialis* (Caballero 1958). COLIMA: Comala: *Didelphis marsupialis* (García-Prieto et al. 2012); La Esperanza: *Didelphis marsupialis* (Miyazaki et al. 1980); ND: *Didelphis virginiana* (Lamothe-Argumedo et al. 1981). DISTRITO FEDERAL: ND: *Didelphis* sp. (Caballero 1937); Chapultepec: *Didelphis marsupialis* (Gutiérrez-Fuster 1966). ESTADO DE MÉXICO: ND: *Didelphis* sp. (García-Prieto et al. 2012). HIDALGO: Tasquillo: *Didelphis* sp. (Caballero 1937). JALISCO: Chamela: *Didelphis marsupialis* (García-Prieto et al. 2012). MORELOS: Reserva Estatal Sierra de Monte Negro: *Didelphis virginiana* (Slava-Araujo 2005). NUEVO LEÓN: San Nicolás de los Garza: *Didelphis virginiana* (García-Prieto et al. 2012). VERACRUZ: Los Tuxtlas: *Didelphis marsupialis*, *Didelphis virginiana*, *Philander opossum* (Cañeda-Guzmán 1997); ND: *Didelphis marsupialis* (Flores-Barroeta 1957).

**Remarks.** We identify these nematodes according to the re-description made by Adnet et al. (2009), who established the number of caudal papillae (ten pairs of button-like papillae, symmetrically ventro-laterally located), as well as the single median papilla at the anterior cloacal lip and four pairs of post-cloacal papillae, as diagnostic traits of this species.

**Family Gnathostomatidae Railliet, 1895**

***Gnathostoma turgidum* Stossich, 1902**

**Site of infection.** Stomach (adult; larvae); liver (sub-adult).

**Present records.** CHIAPAS: Arriaga^†^: *Didelphis* sp. COLIMA: Colima^†^: *Didelphis virginiana*. OAXACA: Soyaltepec^†^: *Didelphis virginiana*. TABASCO: Teapa^†^: *Didelphis marsupialis*. VERACRUZ: Tlacotalpan: *Didelphis virginiana*.

**Specimens deposited.** CNHE 8979–86, 9548–9549.

**Previous records in Mexico.** CHIAPAS: Jaltenengo: *Didelphis marsupialis* (Caballero 1958). COLIMA: Laguna de Amela: *Didelphis virginiana* (García-Márquez 2005). GUERRERO: Laguna de Tres Palos: *Didelphis virginiana* (Monet-Mendoza et al. 2005). JALISCO: Carretera Juntas-Palmas (Puerto Vallarta): *Didelphis virginiana* (Monet-Mendoza et al. 2005); Chamela: *Didelphis virginiana* (see Lamothe-Argumedo et al. 1998). MORELOS: Valle de Amilcingo: *Didelphis virginiana* (Mosqueda-Cabrera 2003). OAXACA: Temascal: *Philander opossum* (Almeyda-Artigas et al. 2010), *Didelphis marsupialis* (Almeyda-Artigas et al. 2000, Oceguera-Figueroa 2002, Mosqueda-Cabrera 2003), *Didelphis virginiana* (Lamothe-Argumedo et al. 1998, Almeyda-Artigas et al. 2000, Mosqueda-Cabrera 2003). SINALOA: Tecualilla: *Didelphis virginiana* (Nawa et al. 2009, Díaz-Camacho et al. 2009). TABASCO: Rancho Mendoza Llergo: *Didelphis marsupialis* (León-Règagnon et al. 2005); Jardín Botánico de la UJAT, Oriente Segunda Sección, Ranchería El Limón, Ranchería Emiliano Zapata, Ranchería José María Pino Suárez, Ranchería La Palma: *Didelphis marsupialis* (Gallegos-Torres 2003). VERACRUZ: Laguna Los Vila, Laguna Novillera: *Didelphis virginiana* (León-Règagnon et al. 2005); Tlacotalpan: *Didelphis virginiana* (Almeyda-Artigas et al. 2000, Pérez-Álvarez et al. 2008), *Didelphis marsupialis* (Pérez-Álvarez et al. 2008).

**Remarks.** The presence of numerous points on the posterior end of cuticular spines at esophagus-intestine junction level, the body size, and the lack of spines in the posterior region of body, constitutes the diagnostic traits of this species in accordance with Bertoni-Ruiz et al. (2011).

**Family Gongylonematidae Hall, 1916**

***Gongylonema* sp.**

**Site of infection.** Stomach.

**Present records.** CHIAPAS: Tapachula^†^: *Didelphis virginiana*.

**Specimens deposited.** CNHE 8970.

**Remarks.** Two species of the genus *Gongylonema* are distributed in Mexican didelphids: *Gongylonema mexicanum* (in Chiapas and Veracruz) and *Gongylonema pulchrum* (in Chiapas) (García-Prieto et al. 2012). The specific identification of our specimen was not possible because we collected only one female.

**Family Physalopteridae Railliet, 1893**

***Turgida turgida* Rudolphi, 1819**

**Site of infection.** Stomach.

**Present records.** CAMPECHE: Escárcega^†^: *Didelphis marsupialis*, *Didelphis virginiana*. CHIAPAS: Arriaga^†^: *Didelphis* sp.; Tapachula^†^: *Didelphis* sp.; *Didelphis marsupialis*. COLIMA: Colima: *Didelphis virginiana*. DISTRITO FEDERAL: Pedregal de San Ángel: *Didelphis virginiana*. GUANAJUATO: Irapuato^†^: *Didelphis virginiana*. HIDALGO: Tianguistengo^†^: *Didelphis virginiana*. OAXACA: Soyaltepec^†^: *Didelphis virginiana*. PUEBLA: Coapan^†^: *Didelphis virginiana*. TABASCO: Teapa: *Didelphis marsupialis*; Villahermosa: *Didelphis virginiana*. VERACRUZ: Los Tuxtlas: *Didelphis marsupialis*, *Didelphis virginiana*; Tlacotalpan^†^: *Didelphis marsupialis*, *Didelphis virginiana*, *Philander opossum*.

**Specimens deposited.** CNHE 9018–23, 9025–36, 9541–9543.

**Previous records in Mexico.** CHIAPAS: Motozintla: *Didelphis* sp. (Caballero and Zerecero 1944, 388); Tonalá: *Philander opossum* (García-Prieto et al. 2012). COLIMA: Colima: *Didelphis virginiana* (Monet-Mendoza et al. 2005); Comala: *Didelphis virginiana* (Monet-Mendoza et al. 2005), *Didelphis marsupialis* (García-Prieto et al. 2012); Dos Amates: *Didelphis virginiana* (Monet-Mendoza et al. 2005); La Esperanza: *Didelphis marsupialis* (Miyazaki et al. 1980); Madrid: *Didelphis marsupialis* (Miyazaki et al. 1980), *Didelphis virginiana* (Monet-Mendoza et al. 2005); ND: *Didelphis virginiana* (Lamothe et al. 1981). DISTRITO FEDERAL: ND: *Didelphis* sp. (Caballero 1937), *Didelphis marsupialis* (Monsivais-Aguilar 1958); Pedregal de San Ángel: *Didelphis virginiana* (Pacheco-Coronel 2010); Chapultepec: *Didelphis marsupialis* (Gutiérrez-Fuster 1966). ESTADO DE MÉXICO: ND: *Didelphis* sp. (García-Prieto et al. 2012): Tequesquinahuac: *Didelphis virginiana* (Monet-Mendoza et al. 2005). GUERRERO: Carretera Coyuquilla-Zihuatanejo, Coyuquilla: *Didelphis virginiana* (Monet-Mendoza et al. 2005); Carretera Aeropuerto-Ixtapa: *Didelphis virginiana* (García-Prieto et al. 2012); Taxco El Viejo: *Didelphis virginiana* (Monet-Mendoza et al. 2005). HIDALGO: Tasquillo: *Didelphis* sp. (Caballero 1937). JALISCO: Chamela: *Didelphis marsupialis* (García-Prieto et al. 2012). MICHOACÁN: El Hortigal: *Didelphis virginiana* (Monet-Mendoza et al. 2005). MORELOS: Reserva Estatal Sierra de Monte Negro: *Didelphis virginiana* (Eslava-Araujo 2005). NAYARIT: Peñitas: *Didelphis virginiana* (Monet-Mendoza et al. 2005). NUEVO LEÓN: Marín, Monterrey: *Didelphis marsupialis* (García-Prieto et al. 2012). OAXACA: Dominguillo: *Didelphis marsupialis* (see Monet-Mendoza et al. 2005); Nizanda: *Didelphis virginiana* (Monet-Mendoza et al. 2005). VERACRUZ: Los Tuxtlas: *Didelphis marsupialis*, *Didelphis virginiana*, *Philander opossum* (Cañeda-Guzmán 1997); Medellín: *Didelphis marsupialis* (Caballero-Deloya 1969).

**Remarks.** These specimens were identified based on the re-description of this species (Matey et al. 2001). Its diagnostic traits are: the presence of 2 spongelike areas on the inner side of each pseudolabia, and the number of caudal papillae (22).

**Family Trichuridae Railliet, 1915**

***Trichuris didelphis* Babero, 1960**

**Site of infection.** Caecum.

**Present records.** CAMPECHE: Escárcega^†^: *Didelphis virginiana*. CHIAPAS: Arriaga^†^: *Didelphis* sp. COLIMA: Colima^†^: *Didelphis virginiana*. HIDALGO: Tianguistengo^†^: *Didelphis virginiana*. MORELOS: Tepoztlán^†^: *Didelphis virginiana*. YUCATÁN: Mérida^†^: *Didelphis virginiana*; Tzucacab^†^: *Didelphis marsupialis*.

**Specimens deposited.** CNHE 8974–78, 9550–9553.

**Previous records in Mexico.** VERACRUZ: Los Tuxtlas: *Didelphis marsupialis*, *Didelphis virginiana*, *Philander opossum* (Cañeda-Guzmán 1997).

Remarks. Our material was identified based on the original description (Babero 1960). This species is characterised by the size of the spicule (0.47–0.6 mm), by having a spiny sheath, by the size of mature eggs (0.068 × 0.032 mm) and the posterior position of the vulva.

**Capillariinae gen sp.**

**Site of infection.** Lungs.

**Present records.** CAMPECHE: Escárcega: Didelphis marsupialis*, Didelphis virginiana*

**Specimens deposited.** CNHE 9031–2.

**Remarks.** Identification was not possible because only eggs were obtained.

**Family Viannaiidae Neveu-Lemaire, 1944**

***Viannaia viannai* Travassos, 1914**

**Site of infection.** Intestine.

**Present records.** CAMPECHE: Escárcega^†^: *Didelphis virginiana*. CHIAPAS: Arriaga^†^: *Didelphis virginiana*, Didelphis marsupialis*. COLIMA: Colima^†^: *Didelphis virginiana*. OAXACA: Soyaltepec^†^: *Didelphis virginiana*. PUEBLA: Coapan^†^: *Didelphis virginiana*. TABASCO: Teapa^†^: *Didelphis marsupialis*; Villahermosa^†^: *Didelphis virginiana*. VERACRUZ: San Andrés Tuxtla^†^: *Didelphis virginiana*, *Didelphis marsupialis*; Tlacotalpan^†^: *Didelphis marsupialis*, Philander opossum*.

**Specimens deposited.** CNHE 8988–98; 9025–30, 9544–9547.

**Previous records in Mexico.** GUERRERO: Taxco El Viejo: *Didelphis virginiana* (Monet-Mendoza et al. 2005).

**Remarks.** Our specimens were identified following Guerrero (1985). The synlophe of *Viannaia viannai* at mid-body has 3 ventral ridges orientated to left, short spicules (0.133-0.141 mm) and bursal ray arrangement 2-1-2 type.

***Travassostrongylus* sp.**

**Site of infection.** Intestine (Adult).

**Present records.** CHIAPAS: Arriaga^†^: *Didelphis* sp.

**Specimens deposited.** CNHE 8987.

**Remarks.** To date, 12 species of the genus *Travassostrongylus* have been described, all parasitizing New World marsupials; *Travassostrongylus orloffi* Travassos, 1935 is the only species of this genus recorded in Mexico as parasite of *Didelphis marsupialis*; however, the finding of only 8 females make species identification difficult, because taxonomy of this group is based on male characteristics (Scheibel et al. 2014).

## Discussion

As a result of this study, we reported 66 new locality records, 9 new host records, and added one species to the composition of the helminth fauna of the opossums in Mexico: the trematode *Brachylaima didelphus* parasitizing *Didelphis virginiana*, which had not been recorded in this country (see García-Prieto et al. 2012). A total of 21 helminth taxa were obtained from the 3 opossums species analyzed (6 trematodes, 2 cestodes, 10 nematodes and 3 acanthocephalans), all in adult stage, with exception of the larvae of *Gnathostoma turgidum* collected during their migration through the liver of the hosts. The richest helminth fauna among the 3 host species was recorded in *Didelphis virginiana*, (parasitized by 17 species), followed by *Didelphis marsupialis* (11 species) and *Philander opossum* (8 species). The digestive tract had the highest number of helminth species (12 intestinals, 2 in gall-bladder, 2 in caeca, and 3 in stomach); only 2 of the 21 taxa, *Didelphostrongylus hayesi* and Capillariinae gen. sp. were found in another site of infection (lungs). The geographic distribution of the helminth species was heterogeneous. The nematode *Cruzia tentaculata* was the only species found in all localities. Other helminth species were collected from 7 (*Trichuris didelphis*), 8 (*Rhopalias coronatus*) and 9 (*Viannaia viannai*) localities; however, most taxa (12) were found in only one locality.

These data bring the number of taxa parasitizing *Didelphis virginiana*, *Didelphis marsupialis*, and *Philander opossum* to 37, 21 and 20, respectively (García-Prieto et al. 2012). In this work we sampled in 9 previously unstudied localities; nevertheless, 47.2%, 52.4% and 40% of the taxa collected were reported previously from the Virginia opossum, Black-eared opossum and Gray four-eyed opossum, respectively. These species are typical of didelphids in other parts of the Americas (see Alden 1995; Corrêa Gomes et al. 2003; Haverskot and Gardner 2008; Bertoni-Ruiz et al. 2011; Richardson et al. 2014), conforming a group basically represented by the trematode *Rhopalias coronatus*, the acanthocephalan *Oligacanthorhynchus microcephalus* and the nematodes *Cruzia tentaculata*, *Gnathostoma turgidum*, and *Turgida turgida*; these species have been recorded associated to any of the three opossum species in 7,10,15, 9, and 17 Mexican states, respectively. In states where the 3 host species are distributed sympatrically, *Oligacanthorhynchus microcephalus* and *Rhopalias coronatus* are the species more frequently shared between them. On the other hand, the most restricted geographic areas are presented by the trematodes *Amphimerus caudalitestis*, *Brachylaima didelphus*, and *Philandrophilus magnacirrus*, the acanthocephalan *Oncicola luehei*, and the nematodes *Gongylonema* sp., and *Travassostrongylus* sp., which are present exclusively in one locality. In total, the records of this group of mammals come from 20 of the 32 states of the Mexican Republic; however, the geographic information is asymmetrical, because most of the samplings were made in the state of Veracruz (13 sites). Other states, as Campeche and Quintana Roo, have been sampled once. Moreover, most of the species that have been found parasitizing these didelphid species represent point locality records in only one study about its parasites cover states or regions, particularly Los Tuxtlas, Veracruz. However, the host’s collections were made along 13 years, in different year season and with a very distinct sample size (see Cañeda-Guzmán 1997).

Considering only the 27 nominal helminth species recorded to date, the 3 host species shared 12 worm species along the sampled sites in Mexico; 8 were exclusively found in *Didelphis virginiana*, and 2 are specialist to *Philander opossum*. The cestode *Thaumasioscolex didelphidis* and the acanthocephalan *Oncicola luehei* are shared by the 2 species of the genus *Didelphis* but not by *Philander opossum*; the Virginia opossum and the Grey four-eyed opossum shared the digenean *Didelphis proloba* and the nematode *Aspidodera raillieti*, whereas *Didelphis marsupialis* and *Philander opossum* shared only *Philandrophilus magnacirrus*. The helminth fauna of these hosts throughout their range is composed by one group of 20 specialist species, and by *Paragonimus mexicanus*, *Oligacanthorhynchus microcephalus*, *Oncicola luehei*, *Paragonimus gethi*, *Aspidodera raillieti*, *Didelphis longispiculata*, and *Thaumasioscolex minuta* that act as generalist species. Accidental species have not been reported in any of the samples carried out to date in Mexico. At a local scale, both phenomena had been also observed in marsupials of French Guiana (Jiménez et al. 2011; Byles et al. 2013).

The structuring factor of the helminth fauna in the three didelphid species is the diet; most of the helminth species infect these host species through ingestion of eggs, larvae or intermediate hosts. Fifteen of the 27 named helminth species have indirect patterns of transmission (*Thaumasioscolex didelphidis*, *Brachylaima didelphus*, *Brachylaima virginiana*, *Didelphis proloba*, *Amphimerus caudalitestis*, *Paragonimus mexicanus*, *Oligacanthorhynchus microcephalus*, *Paragonimus gethi*, *Oncicola luehei*, *Porrorchis nickoli*, *Gnathostoma turgidum*, *Turgida turgida*, *Gongylonema mexicanum*, *Didelphis longispiculata*, and *Didelphostrongylus hayesi*), five are transmitted directly by eggs ingestion (*Aspidodera raillieti*, *Cruzia americana*, *Cruzia tentaculata*, *Trichuris didelphis*, *Trichuris minuta*) and for *Philandrophilus magnacirrus*, *Rhopalias baculifer*, *Rhopalias coronatus*, *Rhopalias macracanthus*, *Rhopalias caballeroi*, *Viannaia didelphis* and *Viannaia viannai*, the life cycle is unknown (Table 2). This result is in agreement with the generalist lifestyles and diets of the three species of opossums (Krause and Krause 2006), that exposed them to the same parasite species; local differences in composition and abundance of helminth species could be related to local availability of parasites (or their intermediate hosts), as well as to the compatibility among host and helminth species, as has been showed by Cañeda-Guzmán (1997) and Jiménez et al. (2011).

**Table 2.** Life cycles of the helminth species collected in the present study.

Phylum	Taxa	Cycle/ Intermediate host	Reference
**Platyhelminthes** **Trematoda**	*Amphimerus* spp.	Heteroxenous/fish	Yamaguti (1975)^‡^
	*Brachylaima* spp.	Heteroxenous/snail	Yamaguti (1975)^‡^
	*Philandrophilus magnacirrus*	Unknown	
	*Rhopalias* spp.	Unknown	
**Cestoda**	*Thaumasioscolex didelphidis*	Heteroxenous/crustaceans	Scholz (1999)^‡^
**Acanthocephala**	*Oligacanthorhynchus microcephalus*	Heteroxenous/millipede	Richardson (2006)
	*Oncicola luehei*	Heteroxenous/insects, crustaceans	Kennedy (2006)
	*Porrorchis nickoli*	Heteroxenous/insects, crustaceans	Kennedy (2006)
**Nematoda**	*Aspidodera raillieti*	Monoxenous/eggs ingestion	Jiménez et al. (2011)
	*Cruzia* sp.	Monoxenous/eggs ingestion	Anderson (2000)
	*Didelphostrongylus hayesi*	Heteroxenous/snails	Prestwood (1976)
	*Gnathostoma* sp.	Heteroxenous/copepods	Kifune et al. (2004)
	*Gongylonema* sp.	Heteroxenous/insects	Anderson (2000)
	*Turgida turgida*	Heteroxenous/insects	Anderson (2000)
	*Trichuris* spp.	Monoxenous/eggs ingestion	Anderson (2000)
	*Viannaia* spp.	Unknown	

‡ Particular life cycle unknown; data obtained at supra-specific level.

The data obtained in this study came from 68 opossums collected from 18 localities (nine not previously sampled for helminths); however, the helminth fauna of each didelphid species showed a stable taxonomic composition with respect to previously sampled sites. Only one species of trematode not previously found in this group of hosts in the country was added to their parasitological record as results of our samples. In spite of the reduced scope of our samplings, this situation suggests that the rate of accumulation of helminth species in the inventory of the 3 species of terrestrial marsupials distributed in the Neotropical portion of Mexico included in this study is decreasing; however, new samplings in the Nearctic portion of this country will probably increase the richness of the helminthological inventory of this group of mammals.
